# The Effects of Cold Water Immersion and Active Recovery on Molecular Factors That Regulate Growth and Remodeling of Skeletal Muscle After Resistance Exercise

**DOI:** 10.3389/fphys.2020.00737

**Published:** 2020-06-30

**Authors:** Jonathan M. Peake, James F. Markworth, Kristoffer Toldnes Cumming, Sigve N. Aas, Llion A. Roberts, Truls Raastad, David Cameron-Smith, Vandre C. Figueiredo

**Affiliations:** ^1^Queensland University of Technology, School of Biomedical Sciences, Institute of Health and Biomedical Innovation, Brisbane, QLD, Australia; ^2^Sport Performance Innovation and Knowledge Excellence, Queensland Academy of Sport, Brisbane, QLD, Australia; ^3^Liggins Institute, The University of Auckland, Auckland, New Zealand; ^4^Department of Molecular and Integrative Physiology, University of Michigan, Ann Arbor, MI, United States; ^5^Norwegian School of Sport Sciences, Oslo, Norway; ^6^School of Human Movement and Nutrition Sciences, The University of Queensland, Brisbane, QLD, Australia; ^7^School of Allied Health Sciences, Griffith University, Southport, QLD, Australia; ^8^Agency for Science, Technology and Research (A^∗^STAR), Brenner Centre for Molecular Medicine, Singapore, Singapore; ^9^Center for Muscle Biology, University of Kentucky, Lexington, KY, United States

**Keywords:** exercise, recovery, cryotherapy, extracellular matrix, adaptation, atrogenes

## Abstract

Regular postexercise cooling attenuates muscle hypertrophy, yet its effects on the key molecular factors that regulate muscle growth and remodeling are not well characterized. In the present study, nine men completed two sessions of single-leg resistance exercise on separate days. On 1 day, they sat in cold water (10°C) up to their waist for 10 min after exercise. On the other day, they exercised at a low intensity for 10 min after exercise. Muscle biopsies were collected from the exercised leg before, 2, 24, and 48 h after exercise in both trials. These muscle samples were analyzed to evaluate changes in genes and proteins involved in muscle growth and remodeling. Muscle-specific RING finger 1 mRNA increased at 2 h after both trials (*P* < 0.05), while insulin-like growth factor (IGF)-1 Ec, IGF-1 receptor, growth arrest and DNA damage-inducible protein 45, collagen type I alpha chain A, collagen type III alpha chain 1, laminin and tissue inhibitor of metallopeptidase 1 mRNA increased 24−48 h after both trials (*P* < 0.05). By contrast, atrogin-1 mRNA decreased at all time points after both trials (*P* < 0.05). Protein expression of tenascin C increased 2 h after the active recovery trial (*P* < 0.05), whereas FoxO3a protein expression decreased after both trials (*P* < 0.05). Myostatin mRNA and ubiquitin protein expression did not change after either trial. These responses were not significantly different between the trials. The present findings suggest that regular cold water immersion attenuates muscle hypertrophy independently of changes in factors that regulate myogenesis, proteolysis and extracellular matrix remodeling in muscle after exercise.

## Introduction

Athletes commonly use cold water immersion to recover after intense exercise, based on the belief that it provides physiological benefits that expedite return to training and competition. However, mounting evidence indicates that when used regularly, cold water exercise can diminish long-term gains in strength and muscle mass after strength training (Peake, 2020). Research into the mechanisms responsible for this effect has revealed that acute cold water immersion after resistance reduces or interferes with several important acute processes and pathways that stimulate muscle hypertrophy, including: muscle protein synthesis, the expression of genes that regulate intracellular amino acid transport, satellite cell proliferation, phosphorylation of kinases in the mTOR and p38-MNK1-eIF4E signaling pathways, and ribosomal DNA transcription (Roberts et al., 2015; Figueiredo et al., 2016; Fyfe et al., 2019; Fuchs et al., 2020). Regular cold water immersion may also attenuate chronic changes in heat shock proteins, while also activating factors responsible for catabolism in muscle [e.g., Forkhead box O (FoxO)] (Fyfe et al., 2019).

Cold water immersion could also influence long-term gains in strength and muscle mass through other mechanisms. In addition to the mTOR and p38-MNK1-e1F4E pathways, muscle growth is regulated through the IGF-1−PI3K−Akt pathway (Schiaffino et al., 2013). The activity of these growth-related pathways in skeletal muscle is balanced by the myostatin−Smad3 and ubiquitin−proteasome pathways, which promote muscle proteolysis (Schiaffino et al., 2013). Resistance exercise activates the IGF-1−PI3K−Akt, myostatin−Smad3 and ubiquitin−proteasome pathways. The gene expression of the IGF-1 isoforms IGF-1Ea, IGF-1Eb, and IGF-1Ec (also known as mechano growth factor) and the IGF-1 receptor increases acutely in skeletal muscle after resistance exercise (Psilander et al., 2003; McKay et al., 2008; Wilborn et al., 2009; Heinemeier et al., 2013). Among the negative regulators of muscle growth, muscle-specific RING finger (MuRF)-1 mRNA increases acutely (Louis et al., 2007; Drummond et al., 2008; Mascher et al., 2008; Reitelseder et al., 2014; Stefanetti et al., 2014; Zak et al., 2018), as does the expression of genes encoding various proteins in the ubiquitin−proteasome pathway (Mahoney et al., 2008). By contrast, myostatin expression decreases (Louis et al., 2007; Deldicque et al., 2008; Drummond et al., 2008; Mascher et al., 2008; Wilborn et al., 2009; Zak et al., 2018). Acute changes in the expression of atrogin-1 (also known as muscle atrophy F-box or F-box protein 32) (Louis et al., 2007; Deldicque et al., 2008; Mascher et al., 2008; Manini et al., 2011; Nader et al., 2014; Reitelseder et al., 2014; Stefanetti et al., 2014; Zak et al., 2018) and FoxO (Louis et al., 2007; Manini et al., 2011; Reitelseder et al., 2014; Stefanetti et al., 2014; Zak et al., 2018; Fyfe et al., 2019; Fuchs et al., 2020) in skeletal muscle after resistance exercise are more variable and time-dependent. The gene expression of growth arrest and DNA damage-inducible protein (GADD45) also increases acutely in skeletal muscle after eccentric exercise (Chen et al., 2003; Mahoney et al., 2008) and endurance exercise (Romero et al., 2016). GADD45 plays a role in both muscle growth (Carson et al., 2002) and atrophy (Ebert et al., 2012).

Together with alterations in these growth factors and so-called “atrogenes” (that mainly regulate protein content in muscle fibers), exercise also induces remodeling of the extracellular matrix (ECM) in skeletal muscle. Exercise stimulates mRNA expression of collagen type I alpha 1 chain, collagen type III alpha 1 chain, matrix metalloproteinases, tissue inhibitor of metallopeptidase, tenascin C mRNA and protein in skeletal muscle (Crameri et al., 2004, 2007; Mikkelsen et al., 2011; Hoier et al., 2012; Heinemeier et al., 2013; Hyldahl et al., 2015). As a result of these changes, collagen formation increases in skeletal muscle after exercise (Crameri et al., 2004; Miller et al., 2005) supporting the repair and growth of new myofibers (Mackey and Kjaer, 2017).

Two animal studies have reported that icing attenuates (Vieira Ramos et al., 2016) or delays (Takagi et al., 2011) the expression of genes or proteins involved in muscle remodeling after injury. Two recent human studies have demonstrated that cold water immersion reduced the expression of phosphorylated FoxO1^Ser256^ (but not the expression of phosphorylated FoxO3a^Ser253^) (Fyfe et al., 2019) whereas it did not alter mRNA expression of FOXO1, MuRF1 or atrogin-1 in muscle after resistance exercise (Fuchs et al., 2020). Some gaps and inconsistencies therefore exist in the literature about how cryotherapies such as icing or cold water immersion influence factors involved in muscle remodeling after exercise and/or muscle injury.

In the present study, we compared how acute cold water immersion and active recovery influence the expression of growth factors, ubiquitin−ligases, myostatin, FoxO and ECM genes and proteins in skeletal muscle after resistance exercise. To achieve this objective, we analyzed muscle samples that we collected as part of a larger study (Roberts et al., 2015). We hypothesized that cold water immersion would attenuate the expression of growth factors and ECM genes/proteins, and enhance the expression of ubiquitin ligases and FoxO3a.

## Materials and Methods

### Ethical Approval

All participants were informed of the requirements and potential risks of the study before providing their written informed consent. The experimental procedures met the standards described in the Declaration of Helsinki, and were approved by the Human Research Ethics Committee of the University of Queensland.

### Experimental Design

We have reported details and the characteristics of the participants of this study elsewhere (Roberts et al., 2015). Nine young, healthy and physically active men (mean ± SD age 22.1 ± 2.2 years, height 1.80 ± 0.06 m, body mass 83.9 ± 15.9 kg) volunteered to participate in the study. The inclusion criteria for the study were that participants were healthy, non-smokers with at least 12 months of experience in strength training, and were not taking any regular medication (e.g., analgesic or anti-inflammatory drugs) that could influence signaling pathways in skeletal muscle. The experimental design comprised a randomized, cross-over study in which the participants performed bouts of single-leg resistance exercise on two separate occasions. After one session, the participants immersed their lower body in cold water for 10 min, while after the other session they exercised at a low intensity for 10 min. The order of these two trials was randomized and counterbalanced among the participants. Muscle biopsies were collected from *vastus lateralis* of the exercised leg before, 2, 24, and 48 h after each exercise session.

#### Strength Exercises

The exercise protocol for the two experimental trial was the same, and involved a combination of lower-body exercises (e.g., 45° leg press, single leg squats, knee extensions and walking lunges). These exercises were performed at loads of 8, 10, and 12 repetition maximum. All strength training was supervised, and was performed in a gymnasium in which the ambient temperature was 23–25°C.

#### Recovery Therapies

In the cold water immersion trial, the participants sat in an inflatable bath (iCool iBody, iCool, Miami, Australia) for 10 min with both legs immersed in water up to the waist. Water was circulated continuously and was maintained at 10.3 ± 0.5°C using a circulatory cooling unit (iCool LITE, iCool). In the active recovery trial, the participants exercised for 10 min active recovery at a self-selected low intensity (36.6 ± 13.8 W) on a stationary cycle ergometer (Wattbike, Nottingham, United Kingdom). To restrict any re-warming following cold water immersion or cooling following active recovery, the participants were prevented from showering or bathing for at least 2 h after the recovery therapies.

#### Muscle Tissue Collection

Muscle biopsies were collected from the mid portion of the vastus lateralis of one leg before exercise and again at 2, 24 and 48 h after each exercise trial. Pre-exercise and 2 h post-exercise biopsies were obtained from the same site on the leg. For the pre-exercise biopsy, the biopsy needle was inserted in a distal direction, whereas for the 2 h biopsy, the needle was inserted in a proximal direction. This method ensured a distance of at least 3 cm between the two biopsy sites. Biopsies at 24 and 48 h were collected from separate sites, each ∼3 cm proximal from the previous site. After the muscle biopsies were taken, the muscle tissue was rinsed with 0.9% saline. Any fat, connective tissue or blood was removed before the sample was frozen in isopentane cooled in dry ice. Samples were stored at −80°C until analysis.

#### Control Procedures

We restricted possible variation in training responses by providing standardized nutrition after each training session, and asking the participants not to exercise for 72 h prior to each trial. Before each trial, the participants ate the same meal 2 h before the pre-exercise muscle biopsy. Before each recovery treatment, they were given a drink containing 30 g serve of whey protein isolate. After consuming this drink, they were only permitted to drink water until the 2 h biopsy was collected. At that time, they were given another 30 g of whey protein isolate to drink. The participants were asked not to take supplements of any kind between 4 d before each pre-exercise biopsy, and the 48 h post-exercise muscle biopsy. The participants were encouraged to adhere to their regular diet for 2 days before each experimental trial and until the 48 h muscle biopsy. Dietary intake before and during the first experimental trial was recorded in a food diary and replicated for the second experimental trial. The participants did not exercise until after the 48 h muscle biopsy for each trial.

### Muscle Tissue Analysis

#### RT-PCR

Total RNA was extracted from ∼20 mg of muscle tissue using the AllPrep^®^ DNA/RNA/miRNA Universal Kit (QIAGEN GmbH, Hilden, Germany). cDNA was prepared using High-Capacity RNA-to-cDNA^TM^ kit (Life Technologies, Carlsbad, CA, United States). Messenger RNA (mRNA) was quantified using RT-PCR on a LightCycler 480 II (Roche Applied Science, Penzberg, Germany) using SYBR Green I Master Mix (Roche Applied Science). Table 1 describes the sequences for the primers used in this study. To normalize the RT-PCR data, the geometric mean of three housekeeping genes was used (Vandesompele et al., 2002). Standard and melting curves were performed for each target to determine primer efficiency and single product amplification.

**TABLE 1:** mRNA primer sequences.

Primer	Sequence
*IGF1R* Forward	CTCCTGTTTCTCTCCGCCG
*IGF1R* Reverse	ATAGTCGTTGCGGATGTCGAT
*IGF1Ec (MGF)* Forward	CGAAGTCTCAGAGAAGGAAAGG
*IGF1Ec (MGF)* Reverse	ACAGGTAACTCGTGCAGAGC
*IGF1Ea* Forward	GACATGCCCAAGACCCAGAAGGA
*IGF1Ea* Reverse	CGGTGGCATGTCACTCTTCACTC
*GADD45A* Forward	CGATAACGTGGTGTTGTGCC
*GADD45A* Reverse	GTTGATGTCGTTCTCGCAGC
*GADD45B* Forward	GGGAAGGTTTTGGGCTCTCT
*GADD45B* Reverse	GGTCACCGTCTGCATCTTCTG
*Atrogin1* Forward	AATAAGGAGAATCTTTTCAACAGCC
*Atrogin1* Reverse	TCCATGGCGCTCTTTAGTACTTC
*MuRF1* Forward	GGGACAAAAGACTGAACTGAATAAC
*MuRF1* Reverse	GGCTCAGCTCTTCCTTTACCT
*Myogenin* Forward	GGCCAAACTTTTGCAGTGAATATT
*Myogenin* Reverse	TCGGATGGCAGCTTTACAAACAAC
*Myostatin* Forward	CTACAACGGAAACAATCATTACCA
*Myostatin* Reverse	GTTTCAGAGATCGGATTCCAGTAT
*Collagen type I alpha chain 1* Forward	TGAAGGGACACAGAGGTTTCAG
*Collagen type I alpha chain 1* Reverse	GTAGCACCATCATTTCCACGA
*Collagen type III alpha chain 1* Forward	GAAAGATGGCCCAAGGGGTC
*Collagen type III alpha chain 1* Reverse	TATACCTGGAAGTCCGGGGG
*Laminin subunit beta 1* Forward	TCCGAGACAGGTCACTGCTA
*Laminin subunit beta 1* Reverse	TGACTCCGCAAAGCAACTGT
*Tissue inhibitor of metallopeptidase* Forward	GGAATGCACAGTGTTTCCCTG
*Tissue inhibitor of metallopeptidase* Reverse	GGAAGCCCTTTTCAGAGCCT
*EMC7* Forward	GGGCTGGACAGACTTTCTAATG
*EMC7* Reverse	CTCCATTTCCCGTCTCATGTCAG
*CHMP2A* Forward	CGCTATGTGCGCAAGTTTGT
*CHMP2A* Reverse	GGGGCAACTTCAGCTGTCTG
*C1orf43* Forward	CTATGGGACAGGGGTCTTTGG
*C1orf43* Reverse	TTTGGCTGCTGACTGGTGAT

#### Western Blotting

To measure FoxO3a protein expression, portions of muscle tissue (45−55 mg) were homogenized and separated into cytosolic and nuclear fractions using a commercial kit (ProteoExtract Subcellular Proteome Extraction Kit, Calbiochem, EMD Biosciences, Germany). The purity of the fractions was evaluated using specific markers for the respective fractions [i.e., GAPDH (cytosol and nuclear) and PARP (nuclear)]. Protein concentration in the samples was quantified in triplicate using a kit (DC Protein Microplate assay, Bio-Rad, Hercules, CA, United States), a filter photometer (Expert 96, ASYS Hitech, United Kingdom), and the software provided (Kim, ver. 5.45.0.1, Daniel Kittrich, Prague, Czechia).

Equal amounts of protein were loaded into each well (11 μg for the nuclear fraction and 20 μg for the cytosolic fraction) and were separated by 4−12% SDS-PAGE under denaturizing conditions for 35−45 min at 200 V in cold MES running buffer (NuPAGE MES SDS Running Buffer, Invitrogen, Carlsbad, CA, United States). All samples were analyzed in duplicate. Following gel electrophoresis, the proteins were transferred onto a polyvinylidene fluoride membrane for 90 min at 30 V using an XCell II Blot Module (Thermo Fisher Scientific, Hemel Hempstead, United Kingdom) and NuPAGE transfer buffer (Invitrogen, Carlsbad, CA, United States). Membranes were blocked at room temperature for 2 h in a 5% fat-free skimmed milk and 0.1% Tris–buffered saline with Tween 20 (TBST) (Bio-Rad; Tween-20, VWR International, Radnor, PA, United States; skim milk, Merck, Darmstadt, Germany). Blocked membranes were incubated overnight at 4°C with a primary monoclonal antibody against FoxO3a (Cell Signaling Technology, Danvers, MA, United States), diluted 1:400. After incubation, membranes were washed and incubated with a secondary antibody (anti-rabbit IgG, HRP-linked antibody, Cell Signaling) at room temperature for 1 h. All antibodies were diluted in a 1% fat-free skimmed milk and 0.1% TBST solution. Between stages, membranes were washed in 0.1% TBST. Bands were visualized using an HRP detection system (Super Signal West Dura Extended Duration Substrate, Thermo Scientific/Pierce Biotechnology). Chemiluminescence was measured using a ChemiDoc MP System (Bio-Rad Laboratories), and band intensities were calculated with molecular imaging software (Image Lab, Bio-Rad Laboratories). All samples were analyzed in duplicate, and mean values were used for statistical analyses.

To measure ubiquitin protein expression, ∼25 mg of tissue were homogenized in RIPA lysis buffer (Millipore, Temecula, CA, United States) composed of Halt^TM^ protease and phosphatase inhibitor cocktail (Thermo Scientific, Waltham, MA, United States). Samples were centrifuged, and protein concentration of the supernatant was measured (Pierce^TM^ BCA Protein Assay Kit, Thermo Scientific). Equal amounts of protein were boiled in 4 × Laemmli buffer; 20 μg of protein were separated by SDS-PAGE and then transferred to PVDF membranes (Bio-Rad Laboratories, Inc., Hercules, CA, United States) using a semidry *Trans-*Blot Turbo^TM^ device (Bio-Rad). The membranes were blocked using 5% bovine serum albumin (BSA) solution in Tris–buffered saline with 0.1% Tween 20 (TBST) for a period of 1 h. Following this step, they were incubated with primary antibodies for ubiquitin (Santa Cruz Biotechnology) diluted 1:1000 in 5% BSA in TBS-T. The membranes were then washed in TBST and incubated with anti-mouse secondary antibodies (Jackson ImmunoResearch Laboratories, West Grove, PA, United States) linked to horseradish peroxidase (1:5000) for 1 h at room temperature. The membranes were exposed on a ChemiDoc image device (Bio-Rad) using enhanced chemiluminescence reagent (ECL Select kit; GE Healthcare Ltd., Little Chalfont, United Kingdom). The staining intensity of the whole lane was measured using ImageJ (NIH, Bethesda, MD, United States), but the bottom of the membrane (∼10 kDa) was excluded to avoid measuring free ubiquitin. Ubiquitinated protein was normalized to GAPDH to ensure equal loading.

#### Immunohistochemistry Staining

Muscle sections were cut to 10-μm thickness in a cryomicrotome (CM 1860 UV, Leica, Nussloch, Germany) set at −20°C, before mounting the sections on microscope slides (Superfrost Plus, Thermo Scientific, Waltham, MA, United States). Muscle sections were fixed in 2% paraformaldehyde for 5 min followed by 10 min permeabilization in 0.2% triton X-100 in PBS. Sections were then blocked in 2% BSA and 5% goat serum in PBS for 60 min at room temperature. Primary antibodies against tenascin C (MA5-16086, Thermo Scientific, Rockford, IL, United States; 1:100) and dystrophin (Ab15277, Abcam, Cambridge, United Kingdom; 1:500) were diluted in 2% BSA and incubated over night at 4°C. The following day, sections were incubated for 60 min at room temperature with appropriate secondary antibodies (A11001, Invitrogen Molecular Probes, Rockford, IL, United States; CF594, Biotium, Fremont, CA, United States; both 1:200) diluted in the blocking buffer. The sections were then covered with ProLong Gold Antifade Reagent with DAPI (P36935, Invitrogen Molecular Probes) and covered with a cover slip. Between stages, sections were washed three times for 10 min in PBS with 0.05% Tween-20. Images of the muscle tissue sections were captured using a camera (DP72, Olympus, Tokyo, Japan) mounted on a microscope (BX61, Olympus) with a fluorescence light source (X-Cite, 120PCQ, EXFP Photonic Solutions Inc., Mississauga, ON, Canada), using a 10 × 0.30 numerical aperture air objective (UPlanFL N, Olympus). To compare the intensity of staining for tenascin C between samples, settings for the microscope, camera and software were fixed. One image was obtained per section, which usually covered most of the sample. By using Fiji imaging software (Schindelin et al., 2012) an optimal threshold for positive staining was set, and used for all images to display percentage area of positive tenascin C staining.

### Statistical Analysis

Prior to statistical analysis, the Shapiro–Wilk test was used to check whether data were normally distributed. Log transformations were applied to data that were not normally distributed. Normally distributed data were analyzed using a 2 × 3 repeated measures ANOVA to calculate time, trial, and time × trial interaction effects. Paired *t*-tests were used to compare changes over time and differences between the trials. Normally distributed data are presented as mean ± SD, whereas log-transformed data are presented as the geometric mean ± 95% confidence interval of the geometric mean. Data that were not normally distributed after log transformation were analyzed using the Friedman’s test. When this test revealed a significant result. Wilcoxon’s signed ranked tests were used to compare any changes over time and differences between the trials. Non-normally distributed data are presented as mean ± interquartile range. The false discovery rate was used to correct for multiple comparisons. The Statistics Package for Social Sciences version 23 (IBM, Armonk, NY, United States) was used to conduct the statistical analysis. Statistical significance for time, trial, and time × trial interaction main effects was set at *P* < 0.05.

## Results

**FIGURE 1:**
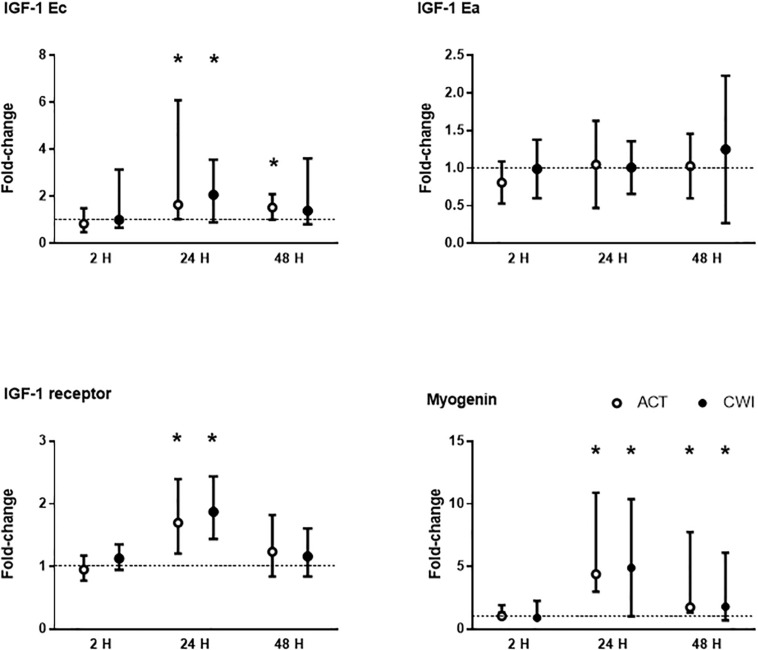
Post-exercise changes in mRNA expression of insulin-like growth factor (IGF)-1 splice variants IGF-1 Ea and IGF-1 Ec (mechano growth factor), IGF-1 receptor and myogenin. Data are presented as changes in the mean ± SD for IGF-1 Ea, and the median ± interquartile range for IGF-1 Ec, IGF-1 receptor and myogenin. *P < 0.05 versus pre-exercise. ACT, active recovery; CWI, cold water immersion. The dotted line represents a nominal pre-exercise value of 1.0 (for comparison).

IGF-1 Ec mRNA expression was higher than pre-exercise 24 h (1.7-fold; *P* = 0.038) and 48 h (1.5-fold; *P* = 0.038) after active recovery, and 24 h (2.1-fold; *P* = 0.038) after cold water immersion (Figure 1). IGF-1 Ea mRNA expression did not change significantly after either trial (*P* = 0.55) (Figure 1). IGF-1 receptor mRNA expression was higher than pre-exercise 24 h after active recovery (1.7-fold; *P* = 0.007) and cold water immersion (1.9-fold; *P* = 0.001) (Figure 1). Myogenin mRNA expression was higher than pre-exercise 24 h after active recovery (4.4-fold; *P* = 0.0039) and cold water immersion (4.9-fold; *P* = 0.0039), and 48 h after active recovery (1.7-fold; *P* = 0.0039) and cold water immersion (1.8-fold; *P* = 0.027). The expression of IGF-1 splice variants, myogenin and IGF-1 receptor did not differ significantly between the trials.

**FIGURE 2:**
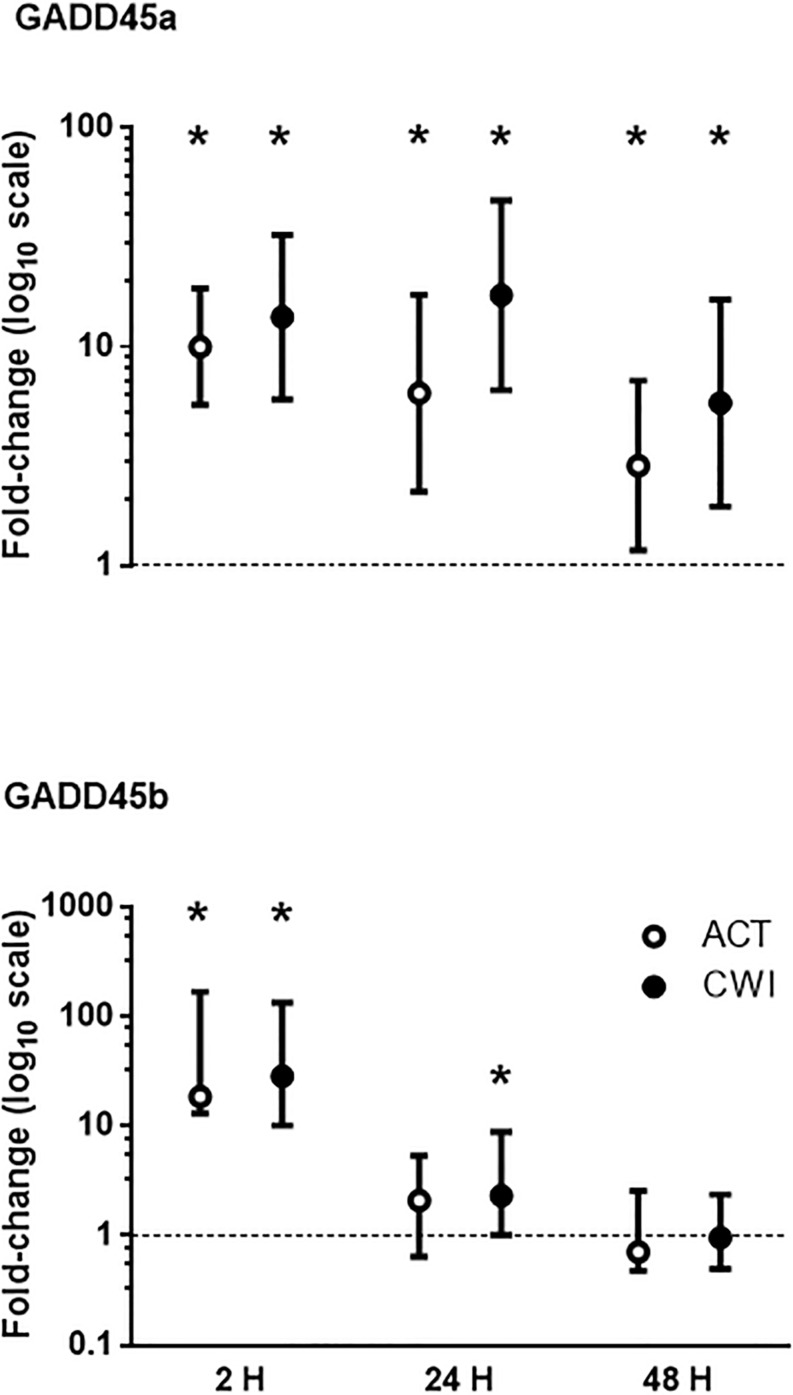
Post-exercise changes in mRNA expression of growth arrest and DNA damage-inducible protein alpha (GADD45a) and GADD45 beta (GADD45b). Data are presented as changes in the geometric mean ± 95% confidence interval of the geometric mean for GADD45A, and the median ± interquartile range for GADD45B. *P < 0.05 versus pre-exercise. ACT, active recovery; CWI, cold water immersion. The dotted line represents a nominal pre-exercise value of 1.0 (for comparison).

GADD45a mRNA expression was higher than pre-exercise 2 h after active recovery (10.0-fold; *P* < 0.001) and cold water immersion (13.7-fold; *P* < 0.001) (Figure 2). It increased further after 24 h, and remained higher than pre-exercise 48 h after both trials. GADD45b mRNA expression was also higher than pre-exercise 2 h after active recovery (18-fold-fold; *P* = 0.008) and cold water immersion (28-fold; *P* = 0.008) (Figure 2). It remained higher than pre-exercise 24 h after cold water immersion (2.3-fold; *P* = 0.028). The expression of GADD45a and GADD45b did not differ significantly between the trials.

**FIGURE 3:**
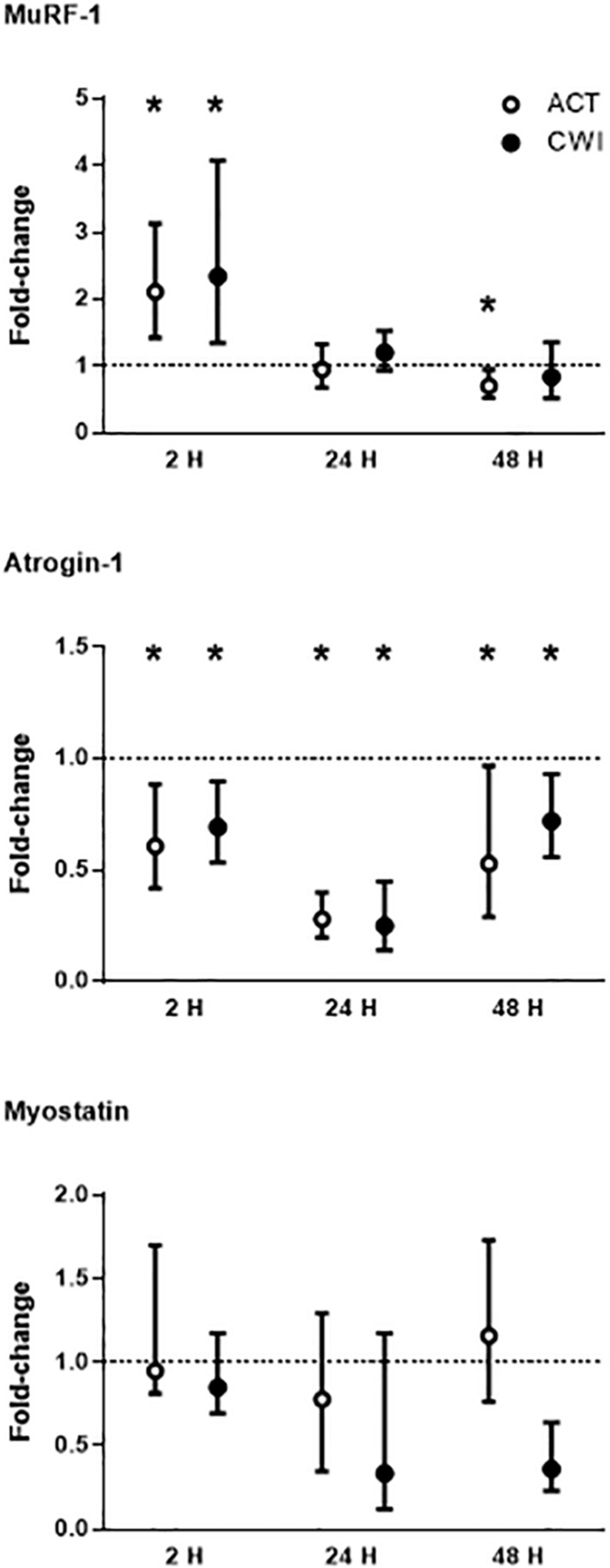
Post-exercise changes in mRNA expression of muscle-specific RING finger 1 (MuRF-1), atrogin-1 and myostatin. Data are presented as the median ± interquartile range. *P < 0.05 versus pre-exercise. ACT, active recovery; CWI, cold water immersion. The dotted line represents a nominal pre-exercise value of 1.0 (for comparison).

MuRF-1 mRNA expression was higher than pre-exercise 2 h after active recovery (2.1-fold; *P* = 0.002) and cold water immersion (2.3-fold; *P* = 0.008) (Figure 3). It was lower than pre-exercise 48 h after active recovery (−5%; *P* = 0.026). Atrogin-1 mRNA expression was lower than pre-exercise 2 h after active recovery (−12%; *P* = 0.015) and cold water immersion (−10%; *P* = 0.012) (Figure 3). It decreased further after 24 h, and remained lower than pre-exercise 48 h after both trials. Myostatin mRNA expression did not change significantly after exercise in either trial (Figure 3; *P* = 0.86 for active recovery; *P* = 0.44 for cold water immersion). The expression of MuRF-1, atrogin-1 and myostatin did not differ significantly between the trials. Ubiquitin protein expression did not change significantly after either trial (*P* = 0.77 for active recovery; *P* = 0.17 for cold water immersion).

**FIGURE 4:**
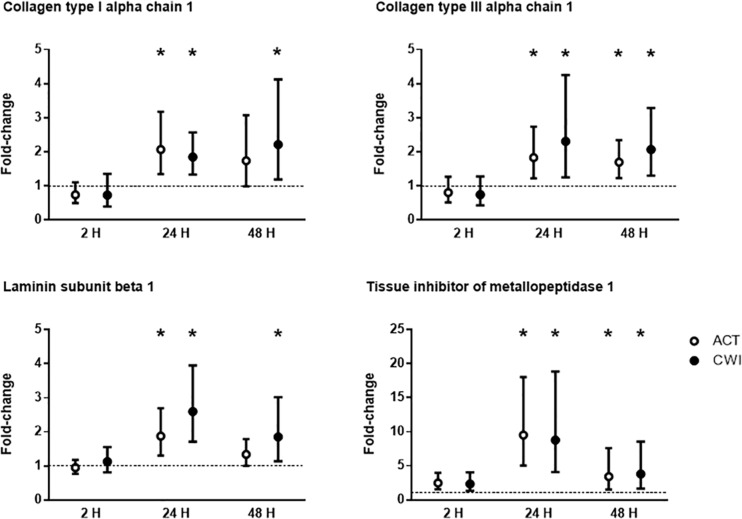
Post-exercise changes in mRNA expression of collagen type I alpha chain 1, collagen type III alpha chain 1, laminin subunit beta 1 and tissue inhibitor of metallopeptidase 1. Data are presented as changes in the median ± interquartile range. *P < 0.05 versus pre-exercise. ACT, active recovery; CWI, cold water immersion. The dotted line represents a nominal pre-exercise value of 1.0 (for comparison).

**FIGURE 5:**
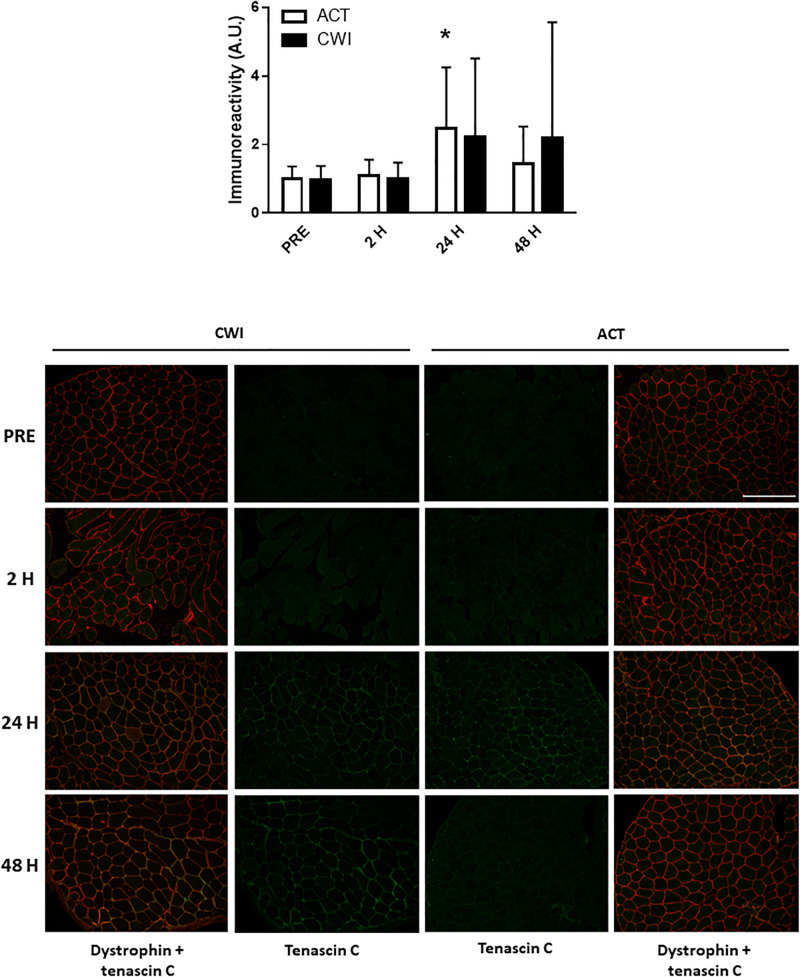
Post-exercise changes in expression tenascin C protein and representative images of tenascin C immunoreactivity in skeletal muscle of one participant. Data are presented as the mean ± SD. *P < 0.05 versus pre-exercise. ACT, active recovery; CWI, cold water immersion. Scale bar represents 200 μm.

Collagen type I alpha chain 1 mRNA expression was higher than pre-exercise 24 h after active recovery (2.1-fold; *P* = 0.004). It was also higher 24 h (1.8-fold; *P* = 0.003) and 48 h (2.2-fold; *P* = 0.019) after cold water immersion (Figure 4). Laminin subunit beta 1 mRNA expression followed a similar pattern of changes compared with COL1A expression (Figure 4). Collagen type III alpha chain 1 mRNA expression was higher than pre-exercise 24 h (1.8-fold; *P* = 0.008) and 48 h (1.7-fold; *P* = 0.005) after active recovery, and 24 h (2.3-fold; *P* = 0.014) and 48 h (2.1-fold; *P* = 0.007) after cold water immersion (Figure 4). Tissue inhibitor of metallopeptidase 1 mRNA expression was higher than pre-exercise 2 h (2.5-fold; *P* = 0.002), 24 h (9.5-fold; *P* < 0.001 and 48 h (3.4-fold; *P* = 0.008) after active recovery. It was also higher than pre-exercise 2 h (2.3-fold; *P* = 0.002), 24 h (8.8-fold; *P* < 0.001) and 48 h (3.8-fold; *P* = 0.008) after cold water immersion (Figure 4). Tenascin C protein expression was higher than pre-exercise 2 h after active recovery (2.5-fold; *P* = 0.022), whereas it did not change significantly after cold water immersion (Figure 5). The expression of ECM genes and tenascin C protein did not differ significantly between the trials.

**FIGURE 6:**
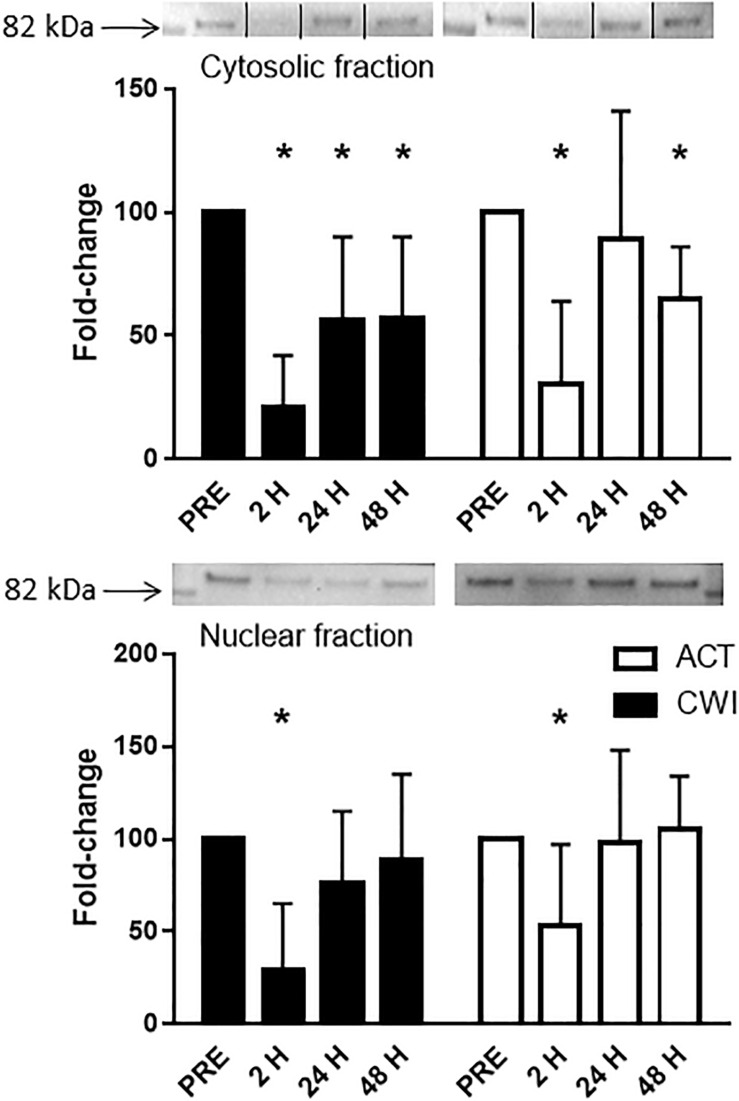
Representative blots and data for expression of FoxO3a in the cytosolic fraction and nuclear fraction isolated from muscle tissue homogenates. Western blotting for FoxO3a in the cytosolic fraction was performed simultaneously on the same membrane for another protein (data published elsewhere (Peake et al., 2017). For the purpose of clarity here, the images for the blots have been cropped (indicated by the lines on the blot) to show only the blots for FoxO3a. Data are expressed as mean ± SD. *P < 0.05 versus pre-exercise. ACT, active recovery; CWI, cold water immersion.

FoxO3a expression in the cytosolic fraction of muscle homogenates was lower than pre-exercise 2 h (0.3-fold; *P* < 0.001) and 48 h (0.7-fold; *P* = 0.001) after active recovery. It was also lower than pre-exercise 2 h (0.2-fold; *P* < 0.001), 24 h (0.4-fold; *P* = 0.005) and 48 h (0.4-fold; *P* = 0.007) after cold water immersion (Figure 6). FoxO3a expression in the nuclear fraction of muscle homogenates was lower 2 h after active recovery (0.5-fold; *P* = 0.029) and cold water immersion (0.3-fold; *P* = 0.001) (Figure 6). The expression of FoxO3a did not differ significantly between the trials.

## Discussion

The present study aimed to compare how cold water immersion and active recovery after acute resistance exercise influence molecular factors that regulate muscle growth and remodeling. Resistance exercise induced the expression of the growth factors IGF-1 Ec and GADD45, the E3 ubiquitin ligase muscle-specific RING finger 1, and various factors involved in ECM remodeling (i.e., collagen type I alpha chain 1, collagen type III alpha chain 1, laminin subunit beta 1, tissue inhibitor of metallopeptidase 1). However, these responses were not significantly different between the cold water immersion and active recovery treatments. The present findings suggest that regular cold water immersion attenuates muscle hypertrophy (Roberts et al., 2015; Fyfe et al., 2019) independently of changes in factors that regulate myogenesis, proteolysis and extracellular matrix remodeling in muscle after exercise.

Consistent with other research (Psilander et al., 2003; McKay et al., 2008; Wilborn et al., 2009; Heinemeier et al., 2013) we found that mRNA expression of IGF-1 receptor and IGF-1 Ec (also known as mechano growth factor) increased in muscle, whereas IGF-1 Ea did not change significantly after resistance exercise (Figure 1). IGF-1 splice variants are proposed to regulate activation, proliferation and differentiation of muscle cells (McKay et al., 2008). We did not find any significant differences in the expression of IGF-1 Ec or IGF-1 receptor mRNA after cold water immersion compared with active recovery. It is unlikely that cold water immersion attenuates chronic muscle adaptation to strength training (Roberts et al., 2015; Fyfe et al., 2019) by reducing IGF-1 signaling pathways in muscle. Our findings contrast with two animals studies reporting that icing either attenuated IGF-1 mRNA expression (Vieira Ramos et al., 2016) or delayed IGF-1 protein expression (Takagi et al., 2011) in muscle after injury. This disparity may reflect differences between the effects of cold water immersion versus icing and the time course of molecular responses in muscle to resistance exercise versus severe muscle trauma.

We also measured mRNA expression of the growth factor GADD45 in muscle after exercise. GADD45 is a small myonuclear protein that translocates to the nucleus of muscle cells in response to cell stress. Here, it modifies myonuclear morphology and activates extensive alterations in the expression of genes that regulate the balance between muscle growth and atrophy (Ebert et al., 2012). GADD45A mRNA remained elevated for the entire recovery period after exercise, while GADD45B mRNA peaked 2 h after exercise (Figure 2), which supports previous research (Chen et al., 2003; Mahoney et al., 2008). GADD45 mRNA expression was not significantly different between the cold water immersion and active recovery trials. The exercise-induced increase in GADD45 in muscle after exercise is intriguing, because it seems to play a dual role in muscle. GADD45 activates many genes associated with muscle atrophy (Ebert et al., 2012). Conversely, it is upregulated in rat muscle following overload-induced hypertrophy (Carson et al., 2002). Also, transfection with plasmids encoding GADD45 in mouse muscle increases myogenin expression (Bongers et al., 2013). GADD45 most likely plays a role in muscle remodeling, but this role may differ under conditions of muscle disuse compared with mechanical loading, including exercise. GADD45 is regulated by histone deacetylase 4 (HDAC4) (Bongers et al., 2013), activating transcription factor 4 (ATF4; also known as CREB2), FoxO and p53 (Kastan et al., 1992; Tran et al., 2002; Ebert et al., 2012). Among these factors, HDAC4 (Russell et al., 2013), FoxO and p53 (Stefanetti et al., 2014) [but not ATF4 (Ogborn et al., 2014)] are activated in muscle after exercise, and may have induced GADD45 mRNA expression in muscle following exercise. Based on the present findings, it would seem that cold water immersion did not influence any of these factors that control GADD45 expression in muscle.

Icing delays muscle regeneration following muscle injury in rats (Takagi et al., 2011) raising the possibility that cryotherapy may activate signaling pathways that inhibit muscle growth, such as the ubiquitin−proteasome pathway. We found that mRNA expression of muscle-specific RING finger-1 increased, whereas atrogin-1 mRNA expression decreased in muscle following resistance exercise (Figure 3). These findings are consistent with other research (Louis et al., 2007; Drummond et al., 2008; Mascher et al., 2008; Manini et al., 2011; Reitelseder et al., 2014; Stefanetti et al., 2014; Zak et al., 2018; Fuchs et al., 2020). However, cold water immersion did not alter the expression of either of these atrogenes. Fuchs et al. also reported no effects of cold water immersion on muscle-specific RING finger-1 and atrogin-1 mRNA expression in muscle between 0 and 5 h after resistance exercise (Fuchs et al., 2020). When comparing the effects of applying cold versus hot thermal pads to muscle after resistance exercise, Zak et al. (2018) observed no significant difference in the expression of these two atrogenes between treatments. It therefore appears unlikely that these ubiquitin-ligases inhibit muscle hypertrophy following repeated cold water immersion (Roberts et al., 2015; Fyfe et al., 2019).

Considering that muscle-specific RING finger-1 and atrogin-1 both promote muscle atrophy (Kang et al., 2017), it is interesting that muscle-specific RING finger-1 increases, whereas atrogin-1 decreases in muscle following resistance exercise. The specific factors that regulate the expression of these two ubiquitin-ligases in muscle during exercise remain to be determined. Likely candidates include FoxO3 (Kang et al., 2017), TNF associated factor (TRAF)-6 (Paul et al., 2012) and the TNF weak inducer of apoptosis (TWEAK)−fibroblast growth factor-inducible 14 (Fn14) system (Hindi et al., 2014). Variation in exercise-induced expression of some of these regulatory factors (Reitelseder et al., 2014; Stefanetti et al., 2014; Raue et al., 2015) may account for the divergent changes in the expression of muscle-specific RING finger-1 and atrogin-1 after exercise that we and others have observed. Muscle-specific RING finger-1 likely influences muscle remodeling after exercise by interacting with (and controlling) the half-life of structural proteins such as troponin, myosin light and heavy chains, and myosin binding protein C (Schiaffino et al., 2013). Atrogin-1 interacts with sarcomeric proteins, the intermediate filaments desmin and vimentin, transcriptional factors, metabolic enzymes and components of translation (Schiaffino et al., 2013). Downregulation of atrogin-1 mRNA expression may result in less degradation of these proteins in muscle after exercise.

In contrast with other research reporting a decrease in myostatin mRNA expression in muscle following resistance exercise (Louis et al., 2007; Deldicque et al., 2008; Drummond et al., 2008; Mascher et al., 2008; Wilborn et al., 2009; Zak et al., 2018), we observed no significant change in myostatin. Myostatin expression in skeletal muscle cells depends (in part) on the expression or activity of follistatin (Lee et al., 2010). The exercise protocol in the present study may not have been sufficiently intense to induce follistatin expression in muscle (Laurentino et al., 2012) and thereby suppress myostatin mRNA expression. Another factor that could account for variation in myostatin responses to exercise is differences in the myostatin splice variants that have been assessed in each study. Interactions between splice variants can influence the expression and activity of myostatin in muscle cells (Shin et al., 2015). There were no differences in mRNA expression of muscle-specific RING finger-1, atrogin-1 or myostatin in response to cold water immersion compared with active recovery.

FoxO proteins are a group of transcription factors that regulate various cellular functions in muscle including energy homeostasis, mitochondrial metabolism, protein breakdown, cell cycle, apoptosis and muscle regeneration (Sanchez et al., 2014). Other research has reported no effect of cold water immersion on gene expression of FoxO1 in muscle after resistance exercise (Fuchs et al., 2020). At the protein level, Fyfe et al. observed that cold water immersion did not alter the expression of phosphorylated FoxO3a^Ser253^ in muscle after resistance exercise (Fyfe et al., 2019). We found that FoxO3a protein levels in cytosolic and nuclear fractions of muscle decreased after exercise, with no significant difference between cold water immersion and active recovery (Figure 6). To our knowledge, this is the first study to compare changes in the protein abundance of FoxO3a in separate subcellular fractions in skeletal muscle after exercise. Others have reported a similar decrease in FoxO3a protein in cytoplasmic and nuclear fractions of C2C12 myotubes treated with IGF-1 (Tong et al., 2009). Tong et al. (2009) also found that IGF-1 increased phosphorylation of FoxO3a at Thr^318/321^ in the cytoplasm. They proposed that in concert with Akt, IGF-1 sequesters and phosphorylates FoxO3a in the cytoplasm, thereby attenuating or blocking ubiquitin−ligase activity (Sandri et al., 2004; Stitt et al., 2004). We did not measure the expression of Akt or phosphorylated FoxO3, so unfortunately, we cannot confirm whether these molecular interactions are also in play in muscle tissue after resistance exercise. It is possible that FoxO is more active and translocates to the nucleus during exercise, whereas after exercise Akt and IGF-1 may suppress this subcellular movement. Regardless, the present findings suggest that cold water immersion did not influence any regulatory factors upstream of FoxO3, such as the IGF-1−PI3K−Akt pathway. Partial support for this notion is that we observed no significant differences in IGF-1 Ec mRNA expression between the trials (Figure 1).

Icing after crush injury increases the proportion of collagen fibers in the extensor digitorum muscle of rats (Takagi et al., 2011). By contrast, freeze injury reduces mRNA expression of collagen I, collagen III and connective tissue growth factor, whereas it does not alter the proportion of collagen fibers in the *tibialis anterior* muscle of mice (Vieira Ramos et al., 2016). To date, no research has investigated the effects of postexercise cooling on ECM remodeling in muscle after resistance exercise. ECM remodeling is an important component of muscle repair and growth. Specifically, in damaged muscle, the ECM provides a scaffold for satellite cells and other stromal cells (e.g., macrophages fibroblasts, endothelial cells, pericytes) to interact with each other to form new muscle fibers (Mackey and Kjaer, 2017). We observed that mRNA expression of collagen type I alpha chain 1, collagen type III alpha chain 1, laminin and tissue inhibitor of metallopeptidase 1 increased in muscle following resistance exercise (Figure 4), which supports the work of others (Crameri et al., 2004, 2007; Mikkelsen et al., 2011; Hoier et al., 2012; Heinemeier et al., 2013; Hyldahl et al., 2015). All of these factors contribute to ECM remodeling, although their individual roles in the reconstruction of myofibers and their matrix requires further research (Mackey and Kjaer, 2017). Staining for tenascin C in muscle also increased after exercise (Figure 5). In the context of ECM remodeling, tenascin-C is believed to disassemble focal adhesion complexes between cells and the matrix, thereby assisting cell survival, motility and tissue repair (Mackey and Kjaer, 2017). The expression of ECM genes and tenascin-C was similar between the cold water immersion and active recovery trials. These findings suggest that the smaller gains in muscle mass and strength after repeated cold water immersion (Roberts et al., 2015) are not likely a result of impaired ECM remodeling.

The strengths of this study include the randomized, cross-over design, the “real-world” resistance training protocol, the inclusion of participants who were training regularly, the pre- and postexercise control procedures, and the breadth of molecular analysis. A limitation is that we did not collect muscle biopsies more than 48 h after exercise. The muscle biopsy procedure is invasive, and the participants in this study provided eight biopsy samples in total. Adding another biopsy sample more than 48 h after exercise would have added to the burden on the participants. Two other studies have reported that mRNA expression of IGF-1, myogenin, atrogin-1 and myostatin in muscle had returned to pre-exercise values by 72−96 h after exercise (Bickel et al., 2005; Deldicque et al., 2008). By contrast, another study reported that mRNA expression of IGF-1Ea, IGF-1Ec, and myogenin remained elevated between 72 and 120 h after exercise (McKay et al., 2008). The time course of changes in factors that regulate muscle growth and remodeling after exercise is therefore variable, and may depend on factors such as the nature of preceding exercise. Another limitation is that we did not include a loading control when performing the Western blots for FoxO3a protein expression. We are confident in our results, however, because we measured total protein concentration in triplicate before loading samples onto the gels, and each sample was analyzed in duplicate.

In summary, although athletes often immerse their body in cold water after intense exercise to expedite return to training and competition, evidence continues to accumulate that when used regularly, this practice can attenuate long-term gains in muscle mass and strength. In the present study, resistance exercise induced robust alterations in various genes and proteins involved in myogenesis, proteolysis and extracellular remodeling in muscle. However, cold water immersion after exercise did not alter the expression of these genes and proteins in muscle between 2 and 48 h after exercise. Putting the present findings into context with other research, regular cold water immersion appears to attenuate adaptations to strength training by suppressing the expression of genes and proteins involved in processes or pathways other than myogenesis, proteolysis and extracellular remodeling.

## Data Availability Statement

The raw data supporting the conclusions of this article will be made available by the authors, without undue reservation.

## Ethics Statement

The studies involving human participants were reviewed and approved by Human Research Ethics Committee of the University of Queensland. The patients/participants provided their written informed consent to participate in this study.

## Author Contributions

JP contributed to the conception, design and interpretation of data for this work, drafted the work, and provided approval for publication of the content. JM contributed to acquisition of data for this work and provided approval for publication of the content. KC, SA, and VF contributed to acquisition of data for this work, drafted the work, and provided approval for publication of the content. LR contributed to the conception and design for this work, and provided approval for publication of the content. TR and DC-S contributed to the conception and design for this work, revised it critically for important intellectual content, and provided approval for publication of the content. All authors contributed to the article and approved the submitted version.

## Conflict of Interest

The authors declare that the research was conducted in the absence of any commercial or financial relationships that could be construed as a potential conflict of interest.
